# Usefulness of Wearable Cameras as a Tool to Enhance Chronic Disease Self-Management: Scoping Review

**DOI:** 10.2196/10371

**Published:** 2019-01-03

**Authors:** Ralph Maddison, Susie Cartledge, Michelle Rogerson, Nicole Sylvia Goedhart, Tarveen Ragbir Singh, Christopher Neil, Dinh Phung, Kylie Ball

**Affiliations:** 1 Institute for Physical Activity and Nutrition School of Exercise and Nutrition Sciences Deakin University Geelong Australia; 2 Department of Epidemiology and Preventive Medicine Monash University Melbourne Australia; 3 Athena Institute for Research on Innovation and Communication in Health and Life Sciences Faculty of Science VU University Amsterdam Netherlands; 4 Department of Medicine Western Health, University of Melbourne Melbourne Australia; 5 Department of Cardiology Western Health Melbourne Australia; 6 Centre for Pattern Recognition and Data Analytics Deakin University Geelong Australia

**Keywords:** eHealth, review, cameras, life-logging, lifestyle behavior, chronic disease

## Abstract

**Background:**

Self-management is a critical component of chronic disease management and can include a host of activities, such as adhering to prescribed medications, undertaking daily care activities, managing dietary intake and body weight, and proactively contacting medical practitioners. The rise of technologies (mobile phones, wearable cameras) for health care use offers potential support for people to better manage their disease in collaboration with their treating health professionals. Wearable cameras can be used to provide rich contextual data and insight into everyday activities and aid in recall. This information can then be used to prompt memory recall or guide the development of interventions to support self-management. Application of wearable cameras to better understand and augment self-management by people with chronic disease has yet to be investigated.

**Objective:**

The objective of our review was to ascertain the scope of the literature on the use of wearable cameras for self-management by people with chronic disease and to determine the potential of wearable cameras to assist people to better manage their disease.

**Methods:**

We conducted a scoping review, which involved a comprehensive electronic literature search of 9 databases in July 2017. The search strategy focused on studies that used wearable cameras to capture one or more modifiable lifestyle risk factors associated with chronic disease or to capture typical self-management behaviors, or studies that involved a chronic disease population. We then categorized and described included studies according to their characteristics (eg, behaviors measured, study design or type, characteristics of the sample).

**Results:**

We identified 31 studies: 25 studies involved primary or secondary data analysis, and 6 were review, discussion, or descriptive articles. Wearable cameras were predominantly used to capture dietary intake, physical activity, activities of daily living, and sedentary behavior. Populations studied were predominantly healthy volunteers, school students, and sports people, with only 1 study examining an intervention using wearable cameras for people with an acquired brain injury. Most studies highlighted technical or ethical issues associated with using wearable cameras, many of which were overcome.

**Conclusions:**

This scoping review highlighted the potential of wearable cameras to capture health-related behaviors and risk factors of chronic disease, such as diet, exercise, and sedentary behaviors. Data collected from wearable cameras can be used as an adjunct to traditional data collection methods such as self-reported diaries in addition to providing valuable contextual information. While most studies to date have focused on healthy populations, wearable cameras offer promise to better understand self-management of chronic disease and its context.

## Introduction

### Background

Noncommunicable diseases, principally cardiovascular diseases, cancer, diabetes, and chronic respiratory diseases, are the leading causes of death in most developed countries, contributing to 60% of all deaths globally [1,2]. However, improved medical management has extended the life expectancy of people with chronic disease. Therefore, treatment goals for many noncommunicable diseases include mitigating exacerbations of the disease to reduce symptoms and prevent hospitalizations [3]. For people with chronic disease, appropriate self-management is critical to maximize health, treatment benefits, and quality of life [4].

Self-management refers to an individual’s engagement in undertaking and managing day-to-day tasks, making and sustaining lifestyle changes, and managing physical symptoms and mental health over the course of an illness [5,6]. This can include a host of activities, such as adhering to prescribed medications, undertaking daily care activities (eg, blood glucose monitoring, self-weighing, rehabilitation exercises, toileting activities), managing body weight (eg, reducing energy intake, increasing physical activity), and managing dietary intake (eg, limiting salt consumption). People with chronic disease need to be supported to engage in and maintain self-management, thus reducing symptoms and empowering them to manage their own health.

Improved self-management has been associated with reduced mortality, hospital admissions, and health care costs [7,8]. For example, a trial of nurse-led education to improve self-management in heart failure demonstrated significant reductions in the relative risk of cardiac (0.59, 95% CI 0.38-0.91) and heart failure-related (0.49, 95% CI 0.27-0.88) hospitalizations [9]. To maximize effective behavioral interventions, efforts must focus on understanding the challenges individuals face in managing the complex demands of their illness and the often multiple and competing conditions. New approaches (with low participant burden and cost) are needed to identify these challenges and effectively tailor interventions to match people’s needs.

The rise of technologies for health care use, such as mobile phones and wearable cameras, offers the potential to facilitate self-management for people with chronic disease [8]. Visual “life-logging” is one such technology. It refers to the use of wearable cameras to digitally capture everyday life activities through first-person point-of-view images [10]. Wearable cameras gather data that accurately reflect the participant’s real-world experiences and environments [11]. Self-reporting of behaviors is difficult and subject to underreporting (eg, dietary intake [12]), overreporting (eg, physical activity [13]), or simply forgetting activities undertaken or food consumed. Wearable cameras can be used to prompt recall and provide health care practitioners with valuable insight into people’s daily behaviors and patterns. This information can assist with prompting subsequent behavior change and developing tailored self-management strategies with patients.

Wearable cameras have been used to assess dietary recall [14] and as an intervention to assist in accurate data collection for a range of activities, such as food purchasing [15], time use [16], sedentary behavior [17], and travel times [18], and to observe behavior changes in early-stage dementia [19]. A recent systematic review to assess the utility of camera images to assist in the assessment of dietary intake found that images can enhance self-report by revealing unreported foods and identify misreporting errors not captured by traditional methods alone [12]. While the feasibility of collecting and analyzing images is well documented across a range of behaviors [14,18], the application of this technology to better understand and augment self-management in people with chronic disease has yet to be investigated and should be considered [20]. For example, wearable cameras could be used for behavior change strategies such as increasing awareness and motivation around specific behaviors [20]. Specifically, wearable cameras could monitor the time and contexts in which individuals take prescribed medications or complete self-monitoring activities [20], such as measuring daily body weight. Using camera-assisted recall methods, this approach could facilitate conversations between patients and health care providers to better tailor self-management strategies.

### Objective

The aim of this review was to ascertain in a human population of any age, with or without chronic disease, what is known about the potential use of wearable cameras for assisting with self-management of chronic disease, including self-management practices such as self-weighing and taking medication, as well as capturing lifestyle behaviors associated with chronic disease (eg, physical activity, sedentary behavior, diet, or smoking). To do this, we searched original research articles reporting studies using any methods, as well as review articles and published conference proceedings.

## Methods

### Rationale for a Scoping Review

Given the novelty of using wearable cameras for enhancing self-management, we considered a scoping review appropriate prior to undertaking a systematic review. Scoping reviews involve systematically searching and selecting literature to map key concepts and summarize a range of evidence to convey the breadth and depth of a field [21,22]. These reviews can be used to examine the extent, range, and nature of research activities, determine the value in undertaking a full systematic review, summarize and disseminate research findings, or help identify gaps in the research literature [23]. Following current guidelines for conducting scoping reviews [21,23,24], once we identified the research question, we proceeded to (1) identify relevant studies, (2) select studies, and (3) collate, summarize, and report the results.

### Identification of Relevant Studies

We conducted a comprehensive electronic literature search in July 2017 in the following databases: PsycINFO, MEDLINE, CINAHL, and SPORTDiscus (all through EBSCO), EMBASE (through OVID), Web of Science, ProQuest, ACM Digital Library, and Cochrane Library. We combined Medical Subject Headings and free terms to search for focused articles. We used a search string including the following search terms and derivatives for each database (see Multimedia Appendix 1 for the full search strategy): (1) wearable camera* OR life-logging OR SenseCam OR Narrative Clip OR GoPro OR Google Glass AND (2) chronic disease OR lifestyle OR lifestyle modification OR rehabilitation OR diet OR physical activity OR medication adherence OR fluid restriction OR smoking.

We also scanned the reference lists of records identified by the search for additional studies that met our inclusion criteria. For the purpose of this review, we were interested in studies that (1) used wearable cameras to capture one or more modifiable lifestyle risk factors associated with chronic disease (eg, physical activity, sedentary behavior, diet, or smoking), (2) used wearable cameras capture typical self-management behaviors (eg, taking medication, self-weighing), or (3) involved a population group with chronic disease (eg, cardiovascular disease, diabetes, respiratory disease). Wearable cameras can capture images actively and passively [25]; in this review, we excluded studies that only used active image capture, were constrained in context (eg, laboratory settings), or collected data for less than 1 day. We also excluded studies using videos due to concerns around battery life constraints (reducing wear time) and the increased difficulty of annotating and coding video images. The search strategy was not limited by study design or year, and it included conference proceedings and full articles but was limited to articles written in English. As a systematic literature review on the effect of wearable cameras on memory disorders had been conducted recently [26], we excluded articles that used wearable cameras in managing forms of dementia, such as Alzheimer disease.

### Study Selection

**Figure 1:**
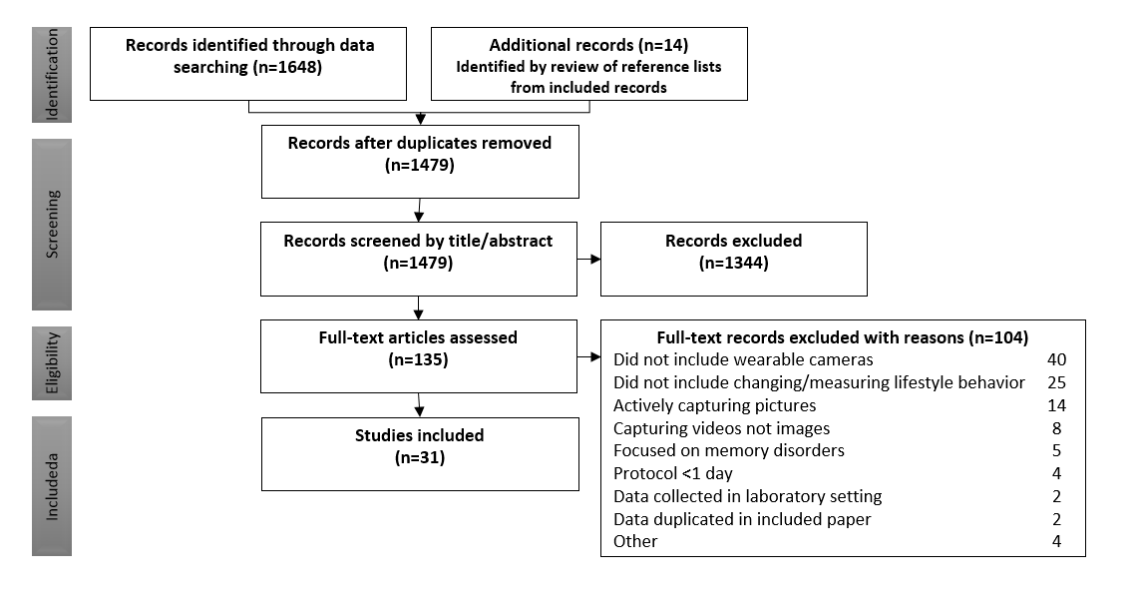
Preferred Reporting Items for Systematic Reviews and Meta-Analyses (PRISMA) flowchart depicting the article selection process.

We imported search results from the databases into the reference software package EndNote version X8 (Clarivate Analytics), which automatically removed most duplicates, with the remaining removed manually. Textbox 1 lists the inclusion criteria for the title and abstract screening. Figure 1 presents a flow diagram leading to the included studies.

**Criteria for article inclusion in the scoping review.**

**Inclusion criteria**

Title-level screening:

- If the title of the article contained the following concepts:
  - Wearable cameras (eg, SenseCam, Narrative Clip, GoPro)
  - Life-logging

Abstract-level screening:

- If the article
  - Reported the use or effect of the wearable cameras or life-logging on a lifestyle behavior or chronic disease *or* measured lifestyle behavior with wearable cameras or life-logging *and*
  - Was written in English *and*
  - Reported qualitative or quantitative findings *and*
  - Passively captured images *and*
  - Measured free-living activities *and*
  - Used the wearable camera for ≥1 day

**Exclusion criteria**

- Articles focused on participants with memory disorders

### Data Extraction

Data extraction was performed by 4 reviewers (NSG, RM, MR, and SC) using a custom data extraction sheet (see Multimedia Appendix 2), which comprised 3 sections; article overview (eg, authors, title, year, country, journal, study design, study aim, camera device, interval of images taken), measuring lifestyle (eg, lifestyle behavior, aim to measure or to change behavior), and study details (eg, sample size and characteristics, intervention duration, presence of a control group, type of image annotation, data analysis, challenges or issues with cameras).

### Summarizing and Reporting the Results

We categorized and described the included studies according to study characteristics, which included the country where the study was undertaken, characteristics of participants (eg, size of sample, age, health status, presence of chronic disease), study design or type, and behaviors measured using wearable cameras (eg, physical activity, dietary intake, sedentary behavior). We also classified studies according to the data collected (primary or secondary) and whether it was a review or discussion article.

## Results

### Study Characteristics

**Figure 2:**
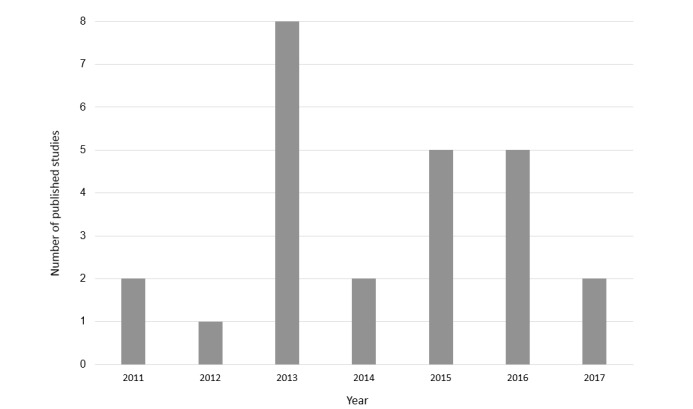
Number of published primary and secondary data studies by year of publication.

Multimedia Appendix 3 [14-17,25,27-46] presents the main results of primary and secondary data collection. Table 1 [11,12,47-50] presents study characteristics of review and discussion studies. Of the 31 studies identified, 22 analyzed primary data and 3 performed a secondary analysis of existing data (Multimedia Appendix 3). These studies were published between 2010 and June 2017, with most primary or secondary data collection studies (22/25, 88%) published since 2013 (Figure 2). Our search strategy also identified 2 discussion articles, 2 reviews, and 2 descriptive articles (Table 1).

Studies undertaking primary or secondary data analysis (n=25) were predominantly feasibility or pilot studies (n=13, 52%) [15,18,25,27-32,34-36,43,46], followed by methodological studies (n=6, 24%) [37,39-41,44] and validation studies (n=4, 16%) [14,33,42,46]. There was 1 randomized controlled trial [38] conducted with acquired brain injury patients where camera images formed part of a health intervention. There was also 1 descriptive study, which described the context of sedentary time in older adults [17]. The majority of studies were conducted in the United States (n=8, 32%) [27,32,35-37,42-44], the United Kingdom (n=6, 24%) [15-17,28,31,45], and Ireland (n=4, 16%) [30,33,39,41]. A total of 3 studies (12%) [29,34,46] were international multicenter studies, while the remaining studies were from New Zealand (n=3, 12%) [14,25,40] and Spain (n=1, 4%) [38].

Of the remaining 6 review or discussion studies (Table 1), 2 reviews focused on dietary assessment [14,47]. One descriptive study presented a research program and a new device for recording food intake [48], while the other described a wrist-worn device for measuring physical activity [49]. The final 2 studies were discussion articles examining the utility of using wearable cameras for assessing lifestyle behaviors (eg, sedentary and nutrition behaviors, television viewing), their challenges, and ethical-related issues [11,50].

Feasibility and pilot studies [15,18,25,27-32,34,36,43,46] focused predominantly on assessing the feasibility of using wearable cameras to capture information on dietary intake or food consumption, physical activity, and sedentary behavior.

**Table 1:** Characteristics of review and discussion studies (n=6).

Study		Design	Camera device	Aim	Findings
**Dietary intake**					
	Boushey, 2016 [47]	Narrative review	Multiple	Overview of image-assisted and image-based methods, including implementation and detail on image-based food records.	Accuracy of dietary assessment was improved. Underreporting was reduced in all included studies.
	Gemming, 2015 [12]	Systematic review	Multiple	Examination of studies that evaluated or validated image-assisted methods of assessing dietary energy intake.	Evidence regarding the validity of image-assisted methods of dietary assessment was limited. Self-reported data could be enhanced with images, providing a primary record of dietary intake to obtain valid estimates of energy intake.
	Sun, 2010 [48]	Descriptive (research program)	Prototype	Description of emerging science for objective methods of dietary assessment.	A research program to automatically record food intake was described. Hardware (camera, reference lights, accelerometer, microphone, global positioning system, and data processor) had been developed; software (enabling privacy protection, video segmentation, food identification, portion size analysis, and nutrient and calorie determinations) was under development.
**Physical activity**					
	Maekawa, 2012 [49]	Descriptive (device)	Wrist-Sense	Description of the implementation of a wrist-worn sensor device.	The camera supplied information on what object the wearer was holding, which related strongly to the activity the wearer was performing.
**Activities of daily living**					
	Doherty, 2013 [11]	Discussion	Multiple	Assessment of the utility of wearable cameras for objectively measuring lifestyle behaviors.	The use of wearable cameras was considered appropriate to understand lifestyle behaviors.
	Loveday, 2016 [50]	Discussion	Multiple	Discussion of the objective measurement of context and illustration of the utility of quantifying context using example data from 3 ongoing studies.	Devices that provide contextual information, such as wearable cameras, location monitors, and proximity sensors, provided researchers with a more comprehensive picture of behavior.

Studies classified as validation studies [14,33,42,46] assessed the validity of wearable cameras either compared with or in combination with other measurement approaches, including self-reported diaries and questionnaires, as well as objective measurement techniques such as accelerometers and doubly labelled water. For the methodological studies [35,37,39-41,44], outcomes focused on testing software or analytic approaches to classify and analyze generated images.

### Sample Characteristics

**Figure 3:**
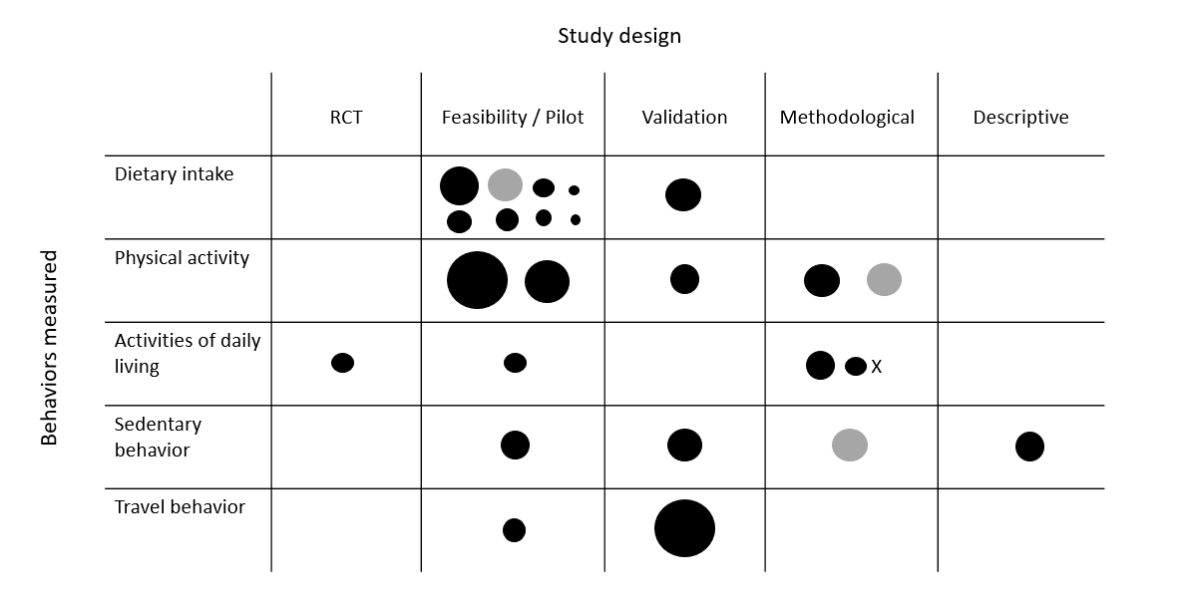
Behaviors measured, study design, and sample size of primary and secondary data collection studies (n=25). Studies are represented by black and gray circles proportional to the study sample size. X denotes 1 study that did not report sample size. Black circles represent studies that used primary data. Gray circles represent studies that used secondary data for analysis. RCT: randomized controlled trial.

Where sample size was relevant and reported (n=24), numbers were generally small (Figure 3): 8 studies (33%) had fewer than 20 participants (minimum of 5) [16,27-29,31,32,38,41], 13 studies (54%) had between 20 and 50 participants [14,15,17,25,30,33,35,37-39,42-44], and only 3 studies (13%) [34,36,46] had more than 50 participants (maximum of 84). Participants were predominantly healthy adults (n=16, 64%) [14,16,17,25,27-29,31,32,34-36,39,41,44,46], with some studies including athletes (n=4, 16%) [30,33,42,43] or school children (n=2, 8%) [15,40]. One study examined physical activity and sedentary time in a sample of older women [37]. No studies identified in our review recruited participants with a chronic disease; however, 1 study included participants with an acquired brain injury [38]. The majority of studies included convenience samples, often recruited through universities; thus, participants were more likely to be well educated with higher socioeconomic status.

### Types and Uses of Wearable Cameras

The SenseCam (subsequently called Vicon Revue, then Vicon Autographer) was the most frequently used brand of wearable camera (n=18, 72%). The remaining studies used a similar technology, worn around the neck on a lanyard or on the wrist. In 22 studies (88%), participants wore a camera for 3 to 7 days; in 1 study, participants wore the cameras for 7 weeks [38]. Studies used wearable cameras to measure, observe, or validate specific behaviors related to dietary intake (n=10, 40%) [14,15,25,27-32,48]; physical activity (n=6, 24%) [33-37,49]; a diverse range of daily activities such as travel, work, and exposure to food marketing (n=5, 20%) [16,38-41]; sedentary behavior (n=4, 16%) [17,42-44]; and travel behaviors such as walking, cycling, or motorized transport (n=2, 8%) [45,46].

Our search found only 1 randomized controlled trial [38], which combined the SenseCam and Actiheart device for goal management training over 7 weeks for 16 people with an acquired brain injury. Results showed that goal management training plus the addition of viewing SenseCam images resulted in greater improvements in cognitive skills compared with goal management training alone.

### Issues Associated With Wearable Cameras

Findings from the studies identified in this review highlighted a variety of technical and personal constraints associated with using this technology. These included person-related factors such as feeling self-conscious while wearing the device, forgetting to put the device on, and privacy and ethical concerns for both the person wearing the device and those whose images were being taken. Technical issues noted included short battery life, challenges in analyzing and classifying very large numbers of images captured by the device, and lack of consistent high-quality images.

### Utility of Wearable Cameras for Self-Management

Notwithstanding the technical issues reported in many studies, most highlighted that wearable cameras offered a feasible and acceptable method for measuring specific behaviors, namely food consumption, physical activity, and sedentary behaviors, and identifying activities of daily living, especially when used alongside more traditional methods of assessment such as logbooks, diaries, and self-recall questionnaires.

Based on this review, the most common use of wearable cameras was for prompting recall of dietary intake and to identify unreported items [14,15,27,28,30]. In these studies, participants were asked to recall their dietary intake using 24-hour dietary recall reported questionnaires [14,30] or interview [28] techniques alone and then again in combination with reviewing SenseCam images. All 3 studies showed that viewing the images increased self-reported energy intake by 12.5% [28], 8% (men) and 6% (women) [14], or 10.7% to 17.7% [30] compared with 24-hour recall alone. These results suggested that a more accurate estimate of total energy intake is possible by combining the SenseCam data with conventional food diaries.

Wearable camera data also offered promise for dietary assessment [25,30,31] and to augment understanding of eating moments or episodes by providing spatial and contextual information [25,29]. Data from 1 earlier validation study in 2013 identified that eating moments could be identified with a high degree of accuracy (89.7%) [32].

The next most common use of wearable cameras was to augment measurement of physical activity-related behaviors, including travel behaviors such as cycling and using public transport [45,46]. Kelly et al [45] tested the feasibility of measuring travel to school in 2012, followed by validating travel data against a travel diary in 2014 [46] with 84 adults using a variety of transportation modes. Both studies found that self-reported journey data were accurate at the group level but imprecise at the individual level.

Combined with traditional measurement techniques (eg, accelerometry), wearable cameras were used to provide context (type and location) for physical activity [33,34] and sedentary behaviors (eg, location, activity undertaken while sedentary) [17,43]. However, data analysis in these areas is still in its infancy, with 3 methodological studies describing the challenges of developing algorithms [37], machine learning techniques [35], and classification techniques [44] for processing wearable camera images.

The complexity of identifying activities of daily living using wearable cameras was made evident by the high number of methodological studies (3 of 5 studies) in this category [39-41]. These studies aimed to identify personal traits [39], everyday activities [41], and children’s exposure to food marketing and other public health issues (eg, tobacco exposure) [40]. These studies analyzed millions of images (from 1.4 million [40] to 3.5 million [39]) using both manual and software-assisted coding methods.

Using camera images to prompt recall during interviews, 1 study demonstrated the feasibility and acceptability of using wearable cameras to reconstruct daily use of time [16]. We found a single randomized clinical trial, which used daily camera images to enhance self-management by people with an acquired brain injury [38]. This study showed that, in addition to goal management training, reviewing SenseCam images in the intervention arm resulted in health improvements (eg, planning, self-monitoring, error detection), both quantitatively (increased effect sizes between groups) and qualitatively (increased engagement). This was the only study to use wearable cameras as a health intervention and highlighted the potential of extending this approach beyond acquired brain injury to other clinical conditions (such as cardiovascular disease, diabetes, and peritoneal dialysis).

## Discussion

### Principal Findings

We conducted this scoping review to ascertain what is known about the use of wearable cameras for self-management by people with a chronic disease and to determine the potential for using this approach for helping people to better manage their chronic disease. We identified 31 studies, with most published since 2013, highlighting the infancy of the field. Based on the available evidence, no studies used wearable cameras for self-management activities, but the searched literature demonstrated that wearable cameras have utility for capturing health-related behaviors and lifestyle risk factors of chronic disease, such as dietary intake, physical activity, and sedentary behavior. Thus far, wearable cameras have been predominantly used to augment existing measurement approaches of these behaviors by assisting with recall of activities (eg, eating, sitting at a computer) or have been used to provide contextual information on these behaviors (eg, walking in a park, or associated behaviors such as sitting at a table eating food). These lifestyle behaviors are important for the prevention and management of many chronic diseases but, as we have demonstrated in this review, wearable cameras have yet to be used by people with existing disease. Furthermore, other than Silva and colleagues’ [26] study of the use of wearable cameras to assist with memory and recall for those affected by Alzheimer disease, there is a paucity of research on the use of wearable cameras for capturing other disease self-management practices such as taking medication, self-weighing, and undertaking home dialysis.

The majority of included studies focused on young healthy individuals, with a subset of studies investigating wearable camera use with athletes [30,33,42,43] and school children [15,40]. One study [37] investigated walking and sedentary time in older women, a group that may be at higher risk of chronic disease. We could identify only 1 study [38] that used the SenseCam as an intervention tool for people with an acquired brain injury, which highlighted the potential of this approach to improve health outcomes of those with an injury or chronic health condition. At the time of our review, we could not identify any further studies investigating the use of wearable cameras in other clinical populations.

In terms of diet, our findings are consistent with a systematic review (included in this review) [12], which assessed the utility of images captured using both handheld devices and wearable cameras for supporting traditional self-report methods or to provide a primary record of dietary intake. Of the 13 included studies in the review by Gemming et al [12], 10 used handheld devices, while 3 studies used wearable cameras. Findings from the systematic review showed that images enhanced self-report by revealing unreported foods and identifying misreporting error. In addition, when used as a primary record of dietary intake, images provided valid estimates of energy intake [14,30]. However, both image quality and camera position influenced the quality of data collected, which needs to be considered in future studies involving dietary behaviors [14]. Our scoping review also highlighted studies that investigated the use of wearable cameras for capturing contextual factors and other behaviors associated with food consumption and motivations to eat [25,29].

Privacy and ethical issues associated with captured images need to be considered in future research. Doherty et al [11] argued that this method is useful for observing and understanding participant behavior and could be used as a lifestyle behavior change catalyst; however, only 1 study [38] identified in this review used it for this purpose. Given issues of participant and third-party privacy, ethical frameworks have been developed to guide the use of wearable cameras. One such article [51] presented a research checklist to be used for studies with automated wearable cameras. The checklist addressed informed consent, privacy and confidentiality, nonmaleficence, and autonomy of third parties. Future studies using wearable cameras for self-management in clinical populations need to closely consider these privacy and ethical issues.

Technical issues associated with using wearable cameras present numerous challenges, which need to be considered when undertaking future research. These include issues such as participant compliance and adherence with wearing the device due to factors such as limited battery life and the need to recharge devices, not wanting to wear them, and being self-conscious around others. However, the biggest issues that potentially limit the use of these technologies in their current form relate to lack of image clarity, the difficulty of correctly coding images to reflect specific behaviors, and processing vast amounts of image data [11,41,44]. Of the studies included in this review, 4 developed data algorithms and object classification methodology [37,39,41,44]. As highlighted by these studies, using machine learning techniques, such as deep convolutional neural networks, it is possible to undertake automated image recognition [52,53]. Google has a pretrained model for ImageNet, consisting of a collection 10 million images depicting 1000 object categories. Using this approach, it is possible to determine corresponding probabilities of correct identification of images according to specific labels. The precision of this approach and others, while they are useful, remain variable. Manual processing (reviewing and coding) of images is time and resource intensive. Analysis of images from wearable cameras for summary purposes or for creating viable interventions remains challenging. Thus, the utility of wearable cameras with multiple participants over extended periods of times (eg, 6 months) is unclear. Finally, an ongoing challenge is that many of the devices used in the included studies are no longer available (eg, SenseCam/Vicon and Narrative Clip). In this review, we did not include mobile phone–enabled cameras; however, given their ubiquity, they do offer a viable solution for capturing some activities such as dietary intake, but they do not provide the passive data collection afforded by current wearable camera technologies.

### Strengths and Limitations

This is, to our knowledge, the first scoping review to examine the use of wearable cameras for self-management. We conducted an extensive search of the literature using 9 databases from both health and sports science. We did not impose limits on study design and therefore included a diverse range of studies from over 6 countries. On the basis of the number of published studies identified in our original search, we chose not to extend the search to include gray literature. Nor did we perform a quality assessment, as it is suggested that this is not in the remit of scoping reviews [54]. More recently, a consultation phase has been suggested to be included in scoping reviews [24]. This phase involves formally presenting the findings to knowledge users and community members to gain collective experience, expertise, and knowledge on the chosen subject [24]. While recommended, this approach is not mandatory; thus, we did not include a consultation phase as part of this review.

### Future Research

Findings from this review suggest that there is interest in the use of wearable cameras to assess lifestyle behaviors, particularly diet, physical activity, and sedentary behavior. In its current form, this method has been used to augment existing measurement techniques through validation, reduction of error associated with recall of behaviors, and provision of rich contextual information. The lessons learned from this research are important if we are to better evaluate these behaviors and empower and support patients with self-management.

There appears to be considerable opportunity to use wearable cameras to specifically assess self-management behaviors and to apply this method to a host of clinical conditions. For example, heart failure is a chronic condition, often with a variable clinical course and frequent exacerbations of symptoms [3]. Appropriate self-management is critical to maximize treatment benefits [4]. To maximize effective behavioral interventions, efforts must focus on understanding the challenges individuals face in managing the complex demands of their illness. Wearable cameras could be used to capture the ecological context in which people manage this disease and identify the extent to which people adhere to key self-management practices (eg, taking medication, daily weighing, fluid restriction, salt consumption in foods). If these behaviors could be correctly identified, then more tailored interventions to match people’s needs could be developed. For example, participants with a new diagnosis of heart failure could wear a camera for 1 to 4 weeks. On the patient’s return to an outpatient clinic, a nurse specialist or other health professional could, by using software, review images alongside the individual to identify self-management practices and offer suggestions for improvement. Such an approach could easily be applied to other scenarios, such as peritoneal dialysis or stroke rehabilitation. Future research is needed to determine the feasibility of such an approach, including whether a chronic disease population would be able to wear and maintain the device for the duration of the intervention and, therefore, how a wearable camera would fit into their lives and disease management [55]. We would expect that the simple nature of these devices would not add additional burden to people with chronic disease and their caregivers, but future research is required [56].

### Conclusion

This scoping review highlighted the use of wearable cameras for the assessment of lifestyle-related behaviors (in particular diet and physical activity) among healthy, adult populations; however, none of the studies specifically focused on self-management behaviors by people with chronic disease or in clinical settings. The advanced capabilities of wearable camera technologies, when considered alongside the gap in the evidence base and early findings of the usefulness of cameras in other populations identified here, all point to the promising potential of this approach and the need for further investigation in clinical populations.

## Appendix

Multimedia Appendix 1 Search strategy.

Multimedia Appendix 2 Data extraction form.

Multimedia Appendix 3 Characteristics of primary and secondary data collection studies.
