# Supplementary material for: Usefulness of Wearable Cameras as a Tool to Enhance Chronic Disease Self-Management: Scoping Review
Source: JMIR Mhealth Uhealth. 2019 Jan 3;7(1):e10371. doi: 10.2196/10371 (PMC6682294; doi:10.2196/10371)
Supplement: Multimedia Appendix 1 [file mhealth_v7i1e10371_app1.pdf]

Search Strategy: Usefulness of wearable cameras as a tool to enhance chronic disease self-management: Scoping review.

Embase	Web of science	PsycINFO, Medline, CINAHL, SPORTdiscuss (through EBSCO)	Proquest	Cochrane Library	ACM Digital Library
Wearable cameras.mp	wearable camer*	“wearable camer*”	“wearable camer*”	Wearable cameras	“wearable camera”
Life-logging.mp	Life-logging	Life-logging	Life-logging	Life-logging	“life-logging”
Sensecam.mp	SenseCam	SenseCam	SenseCam	SenseCam	“SenseCam”
Narrative clip.mp	Narrative clip	“Narrative clip”	“Narrative clip”	Narrative clip	“Narrative clip”
GoPro.mp	GoPro	GoPro	GoPro	GoPro	“GoPro”
Google glass.mp	Google Glass	“Google Glass”	“Google Glass”	Google Glass	“Google Glass”
**AND**	**AND**	**AND**	**AND**	**AND**	
exp chronic disease/	("Chronic diseas*"	"Chronic Diseas*"	"Chronic Diseas*"	Chronic disease	“chronic disease”
exp lifestyle/	"life-style behaviour"	life-style	life-style	Lifestyle behaviour	“lifestyle behaviour”
exp lifestyle modification/	("Life-style modification")	"lifestyle modification"	"lifestyle modification"	Lifestyle modification	“lifestyle modification”
exp rehabilitation/	Rehab*	Rehab*	Rehab*	Rehab*	“Rehab*”
exp diet/	Diet*	Diet*	Diet*	Diet	“Diet”
exp exercise/ or exp physical activity/ or exp physical performance	"Physical activ		"Physical activit*" "motor activit* OR exercise	Physical activity	“physical activity”
exp medication compliance/	"Medication Adherence"	“Medication adherence” OR medication compliance	“Medication adherence” OR medication compliance	Medication adherence	“medication adherence”
“Fluid restriction”.mp.					“fluid restriction”
Exp cigarette smoke/ or exp tobacco smoke/	"Fluid restriction"	“Fluid restriction”	“Fluid restriction”	Fluid restriction	“smoking”
