# Supplementary material for: Usefulness of Wearable Cameras as a Tool to Enhance Chronic Disease Self-Management: Scoping Review
Source: JMIR Mhealth Uhealth. 2019 Jan 3;7(1):e10371. doi: 10.2196/10371 (PMC6682294; doi:10.2196/10371)
Supplement: Multimedia Appendix 2 [file mhealth_v7i1e10371_app2.pdf]

Data Extraction Sheet: Usefulness of wearable cameras as a tool to enhance chronic disease self-management: Scoping review.

Paper overview

Authors / Title / Year / Country / Journal / Study Design

Camera device / Interval of images taken

Data collection (primary / secondary)

Study aim

Measuring lifestyle

Lifestyle behaviour (eg. Physical activity, diet etc)

Lifestyle behaviour (measuring / changing)

Identified behaviours

Study details

Population / Sample size / Characteristics

Intervention duration

Control group (y/n)

Type of image annotation (manual / automatic)

Annotation details

Data analysis

% of images that cannot be analysed

Challenges/issues with cameras

Key findings

Other comments / notes
