# Supplementary material for: Usefulness of Wearable Cameras as a Tool to Enhance Chronic Disease Self-Management: Scoping Review
Source: JMIR Mhealth Uhealth. 2019 Jan 3;7(1):e10371. doi: 10.2196/10371 (PMC6682294; doi:10.2196/10371)
Supplement: Multimedia Appendix 3 [file mhealth_v7i1e10371_app3.pdf]

Characteristics of primary and secondary data collection studies (n=25).

Study	Country	Design	Camera device	Duration device worn	Sample size and characteristics	Key findings
Dietary intake						
Arab, 2011 [27]	United States	Primary (feasibility)	Camera-equipped mobile phone	3 independent days	14 healthy adults	Dietary recall was facilitated with automated imaging. Images were deemed helpful by participants, but wearing device was difficult.
Cowburn, 2016 [15]	United Kingdom	Primary (feasibility)	SenseCam	4 days	22 school students (13-15 years of age)	Interviews combined with SenseCam images assisted in identifying unreported items and misreporting errors.
Gemming, 2013 [28]	United Kingdom	Primary (feasibility)	SenseCam	2 days	10 healthy adults	Wearable cameras enhanced 24 hour dietary recall by objectively revealing unreported foods and misreporting errors.
Gemming, 2015 [25]	New Zealand	Secondary (feasibility)	SenseCam	4 days	40 healthy adults	SenseCam images were feasible for enhancing dietary assessment data by objectively assessing context of eating.
Gemming, 2015 [14]	New Zealand	Primary (validation)	SenseCam	4 days	40 healthy adults	SenseCam images enhanced self-report accuracy of dietary recall.
Ng, 2015 [29]	International	Primary (feasibility)	Autographer	1 week	18 participants who were main household shopper	The use of wearable cameras provided high-level overview and spatial data on food-related practices.
O'Loughlin, 2013 [30]	Ireland	Primary (feasibility)	SenseCam	1 day	47 participants including trainee jockeys, Gaelic footballers and physically active university students	Total energy intake was more accurately calculated when SenseCam was used in conjunction with a food diary.
Pettitt, 2016 [31]	United Kingdom	Primary (pilot)	Wearable micro-camera	2 days	6 healthy adults	Combining images from micro-camera with food diaries improved the accuracy of dietary assessment. In addition valuable information on macronutrient intake and eating rate obtained from images.
Thomaz, 2013 [32]	United States	Primary (feasibility)	Standard mobile phone	3 days	5 participants	From 17,575 automatically captured images eating moments were accurately identified with 89.68% accuracy.

Physical activity						
Connor, 2016 [33]	Ireland	Primary (validation)	SenseCam	1 day	30 collegiate Gaelic footballers	Self-recall training diaries were successfully validated with data fused from the SenseCam and accelerometer.
Doherty, 2013 [34]	International	Primary (feasibility)	SenseCam	3 days	52 university workers	Wearable cameras successfully complemented accelerometer data to objectively identify behavioral type and context information across different activity episodes.
Ellis, 2013* [35]	United States	Primary (methodological)	SenseCam	3-4 days	40 healthy adults	Machine learning techniques for data classification has improved accuracy when trained on free-living data compared to training on controlled or lab data.
Nebeker, 2016 [36]	United States	Primary (feasibility)	SenseCam	1 week	82 participants	Survey of participants wearing SenseCam found primary concern was device comfort and privacy.
Rosenberg, 2017 [37]	United States	Secondary (methodological)	SenseCam	1 weeks	39 older women	SenseCam provided “ground truth annotation” to develop free-living algorithms for walking and sedentary time.
Activities of daily living						
Cuberos-Urbano, 2016 [38]	Spain	Primary (RCT)	SenseCam	7 weeks	16 patients with acquired brain injury	Compared to goal management training (GMT) alone, a combination of viewing SenseCam images with GMT resulted in improvements to cognitive skills and quality of life.
Doherty, 2011 [39]	Ireland	Primary (methodological)	SenseCam	Average 15.1 days	33 participants	Successful evaluation of new technique for eliciting personal traits automatically from visual lifelogs.
Kelly, 2015 [16]	United Kingdom	Primary (feasibility)	Autographer	1 day	14 participants	Wearable cameras are feasible and acceptable for assessing the validity of 24 hour time use diaries.
Signal, 2017 [40]	New Zealand	Primary (methodological)	Autographer	4 days	Not stated	Methodology described enabled objective, automated observation of children’s lives in a range of settings. 95% of Autographer images could be coded.

Wang, 2013 [41]	Ireland	Primary (methodological)	SenseCam	7 days (4 participants)	13 participants from research group. Data from 4 participants used for evaluation.	Successful application of an algorithm to detect 16 everyday activities.
Sedentary behavior						
Kerr, 2013 [42]	United States	Primary (validation)	SenseCam	3-5 days	40 adult cyclists	Comparing SenseCam and ActiGraph sedentary behavior data demonstrated a difference of only 30 minutes in daily estimates. Sitting was not always inactive-SenseCam maybe a useful tool to understand health behaviors such as sitting.
Leask, 2015 [17]	United Kingdom	Primary (descriptive)	Vicon Revue	1-7 days	33 participants	The majority of sedentary time in older adults is accumulated at home, in the afternoon, for leisure and in social isolation.
Marinac, 2013 [43]	United States	Primary (feasibility)	SenseCam	1-2 days	28 adult commuter cyclists	SenseCam was feasible to measure type and context of sedentary behavior. Wear-time of the SenseCam impacted the daily estimates of sedentary behavior.
Moghimi, 2014 [44]	United States	Secondary (methodological)	SenseCam	3-5 days	40 university workers who regularly cycle for transportation	Deep learning based classifier techniques applied and performed better than state-of-the-art visual classification methods.
Travel behavior						
Kelly, 2012 [45]	United Kingdom	Primary (feasibility)	SenseCam	1 week	17 adolescents	SenseCam provided feasible technique to measure travel to school behavior. Self-reported journey duration data was accurate at the mean group level, but imprecise at the individual level.
Kelly, 2014 [46]	International	Primary (validation)	SenseCam	3-4 days	84 participants	Self-reported travel diary data valid for journey level and summary of daily travel duration at a group level, but unreliable at the individual level (journey, day and participant).

*denotes conference proceedings
